# New strategy for controlling root-knot nematodes: research on the resistance of tobacco grafting progenies to root-knot nematodes

**DOI:** 10.3389/fpls.2025.1636579

**Published:** 2025-08-28

**Authors:** Qiankun Li, Xingyang Xu, Hanyang Yao, Yunxia Li, Dekai Ning, Jijuan Tian, Xianqi Hu, Yanmei Yang

**Affiliations:** ^1^ College of Plant Protection, Yunnan Agricultural University/State Key Laboratory for Conservation and Utilization of Bio-resources in Yunnan, Kunming, China; ^2^ Technology Center of Kunming Company, Yunnan Tobacco Company, Kunming, China

**Keywords:** tobacco grafted progeny, root-knot nematodes, disease resistance, agronomic traits, defense enzymes

## Abstract

**Introduction:**

To explore the changes in agronomic traits, differences in disease resistance, and related enzymatic mechanisms of tobacco grafted progeny after inoculation with root-knot nematodes (RKNs), and to elucidate their defense responses.

**Methods:**

We used the F1 progeny of tobacco grafts ‘Banqiao B (moderately resistant rootstock) + Honghua Dajinyuan (susceptible scion)’ (BHF1) and ‘G278 (resistant rootstock)+ Honghua Dajinyuan’ (GHF1) as experimental materials, with the scion variety Honghua Dajinyuan (HD) as the control. We conducted a systematic comparison of agronomic traits, disease resistance, and enzymatic characteristics among the materials 90 days post-inoculation with RKNs.

**Results and discussion:**

Agronomic traits did not differ significantly between the grafted progeny and HD. The disease index (DI) of HD and BHF1 was 74.07, indicating susceptibility (S), while GHF1 exhibited a DI of 22.22, indicating moderate resistance (MR). The Soil and Plant Analyzer Development (SPAD) value of GHF1 was significantly higher than that in HD and BHF1. Although superoxide dismutase (SOD) and catalase (CAT) activities in the leaves and roots of GHF1 were comparable to those in HD, the activities of peroxidase (POD), phenylalanine ammonia-lyase (PAL), polyphenol oxidase (PPO), chitinase (CHT), and β-1,3-glucanase (GLU) in the roots were significantly elevated compared to those in the other treatments. Correlation analysis revealed significant negative correlations between the DI and both the SPAD value and the activities of POD, PAL, PPO, CHT, and GLU, suggesting that increased chlorophyll content and enhanced defense-related enzyme activities contributed to the improved resistance of GHF1. GHF1 thus constitutes a valuable germplasm for nematode resistance. These findings provide a foundation for the selection, propagation, and characterization of grafted tobacco progeny and offer new strategies for breeding tobacco cultivars resistant to RKNs.

## 1 Introduction

Plant-parasitic nematodes (PPNs) are obligate biotrophic parasites with a wide host range and exhibit strong environmental adaptability, infecting nearly all crops worldwide (Elling, 2013). According to yield and market data from 2010 to 2013, the average loss caused by these nematodes in the world’s 20 most important crops was 12.6%, equivalent to $215.77 billion (Abd-Elgawad and Askary, 2018). Root-knot nematodes (RKNs) are one of the most destructive PPNs, severely damaging crops such as tobacco, vegetables, and ornamental plants, and are considered a globally significant disease (Jones et al., 2013). Nematode infection decreases crop growth, yield, and quality, and suppresses chlorophyll synthesis (Strajnar et al., 2012). In response to RKN infection, plants activate metabolic pathways involving antioxidant enzymes, phenylpropanoid metabolism enzymes, and pathogenesis-related proteins to strengthen defense responses (Liang et al., 2012). Among these, antioxidant enzymes and phenylpropanoid metabolism enzymes play crucial roles in mediating plant defense responses (Vos et al., 2013; Molinari and Leonetti, 2023; Zhang et al., 2024). After RKN infection, reactive oxygen species (ROS) accumulate in plants, and key enzymes such as superoxide dismutase (SOD), catalase (CAT), and peroxidase (POD) regulate ROS homeostasis (Shetty et al., 2008). Notably, POD activity has been recognized as a key marker of resistance, with resistant cultivars exhibiting significantly higher root POD activity than susceptible ones (Molinari et al., 2024). The phenylpropanoid pathway biosynthesizes diverse secondary metabolites, including phytoalexins, lignin, and phenolic compounds, contributing to disease resistance (Dixon, 2001). Following RKN infection in *Capsicum annuum*, the activities of phenylalanine ammonia-lyase (PAL) and polyphenol oxidase (PPO) in roots and leaves increase significantly, particularly in resistant cultivars (Ganapathy et al., 2016). In addition, chitinase (CHT) and β-1,3-glucanase (GLU) are enzymes involved in direct pathogen degradation. After pathogen invasion, the activity of CHT and GLU in the roots of resistant varieties is markedly elevated compared to susceptible ones (Zhang et al., 2016).

Tobacco (*Nicotiana tabacum* L.) is an important and widely cultivated cash crop in China, extensively utilized in the production of cigarettes, cigars, chewing tobacco, pipe tobacco, and snuff (Sierro et al., 2014). It is also a well-established model organism in molecular biology and is widely used in studies of disease susceptibility in Solanaceae (Xu et al., 2023). Root-knot nematodes are soil-borne pathogens that pose a serious threat to tobacco throughout its life cycle. Four common RKN species have been identified in tobacco, of which *Meloidogyne javanica*, *M. incognita*, and *M. arenaria* are most widespread, while *M. hapla* is limited to temperate regions (Fortnum et al., 2001). Root-knot nematode disease has escalated in severity across major tobacco-producing regions in China (Huang et al., 2020). In Yunnan Province alone, the disease affects over 26,000 hectares, with yield losses of 30-50% (Tong et al., 2022). While physical, chemical, and biological control methods are partially effective, concerns regarding chemical residues and environmental contamination persist (Ayala-Doñas et al., 2020). Breeding disease-resistant varieties remains the most cost-effective and sustainable strategy (Ayala-Doñas et al., 2020). Grafting, a vegetative propagation technique to improve scion performance, enhances fruit quality, growth, and disease resistance in many Solanaceae and woody species (Albacete et al., 2015), and is increasingly used in plant production and breeding (Song et al., 2016). Theoretically, grafting should not modify genetic information. However, studies show that grafting may induce heritable modifications in plant development, phenotype, and stress responses (Li et al., 2013). Grafting has induced widespread alterations in specific DNA methylation patterns at particular loci, particularly in the scion, and these changes in DNA methylation at specific loci can be inherited by progeny through sexual reproduction (Wu et al., 2018). Soviet fruit breeding expert Michurin, who proposed that rootstocks and scions could exchange genetic material, successfully developed over 300 new fruit varieties using this method (Mudge et al., 2009). More recently, grafting has been shown to facilitate the transfer of genetic material, such as plasmid DNA, potentially bypassing sexual incompatibility and enabling the development of novel species with unique traits (Stegemann and Bock, 2009; Frank and Chitwood, 2016). In cruciferous vegetables, grafting *Brassica oleracea* var. *acephala* onto purple cabbage produced selfed-progeny with diverse phenotypes, including altered apical meristems, earlier flowering, and modified leaf morphology (Cao et al., 2016). MSAP analysis has revealed extensive variations in DNA methylation patterns among the self-pollinated progeny, with approximately one-third remaining stable over five generations (Cao et al., 2016).

Currently, whether variable traits can be obtained through interspecies or intergeneric grafting remains a globally debated scientific question, extensively studies worldwide. For instance, grafting *Brassica rapa* onto purple cabbage resulted in heritable changes in leaf morphology in the progeny (Cao et al., 2016). Similarly, after grafting Arabidopsis with *MSH1*-induced tomato, the traits of the progeny changed, and these changes were maintained over five generations (Hardik et al., 2020). To date, over one billion vegetable plants (mainly tomatoes, peppers, cucumbers, and watermelons) have been grafted globally to enhance their disease resistance (Lee et al., 2010). While most studies have focused on optimizing grafting techniques, the resistance of grafted progeny to RKNs and the associated defense enzyme responses remain poorly understood. In this study, we used greenhouse pot inoculations to assess resistance, agronomic traits, and defense-related enzyme activities in tobacco grafted progeny 90 days post-RKN infection. The aim was to identify nematode-resistant progeny and evaluate the heritability of graft-induced phenotypic changes for potential application in tobacco production.

## 2 Materials and methods

### 2.1 Test materials

Control T0, Scion of ‘Honghua Dajinyuan’ (HD). Tobacco grafting combinations in the progeny: T1, ‘Banqiao B + HD F1’ (BHF1) and T2, ‘G278 + HD F1’ (GHF1). In the parental generation, rootstock ‘Banqiao B’ exhibited moderate resistance, ‘G278’ showed resistance, while scion ‘HD’ was susceptible to the disease. All tested tobacco seedlings were provided by the Kunming branch of Yunnan Tobacco Company.

*Meloidogyne incognita* used for inoculation was collected from Yuanmou County, Yunnan Province, with tomato (*Solanum lycopersicum* L.) as the host plant. Before inoculation, tomato roots were thoroughly cleaned, cut into 1–2 cm segments, and carefully crushed to extract nematode eggs from root knots. After observation and counting under a stereo-microscope, a suspension of RKN eggs was prepared at a concentration of 500 eggs/mL (Ruanpanun and Somta, 2021).

### 2.2 Experimental design

**Figure 1:**
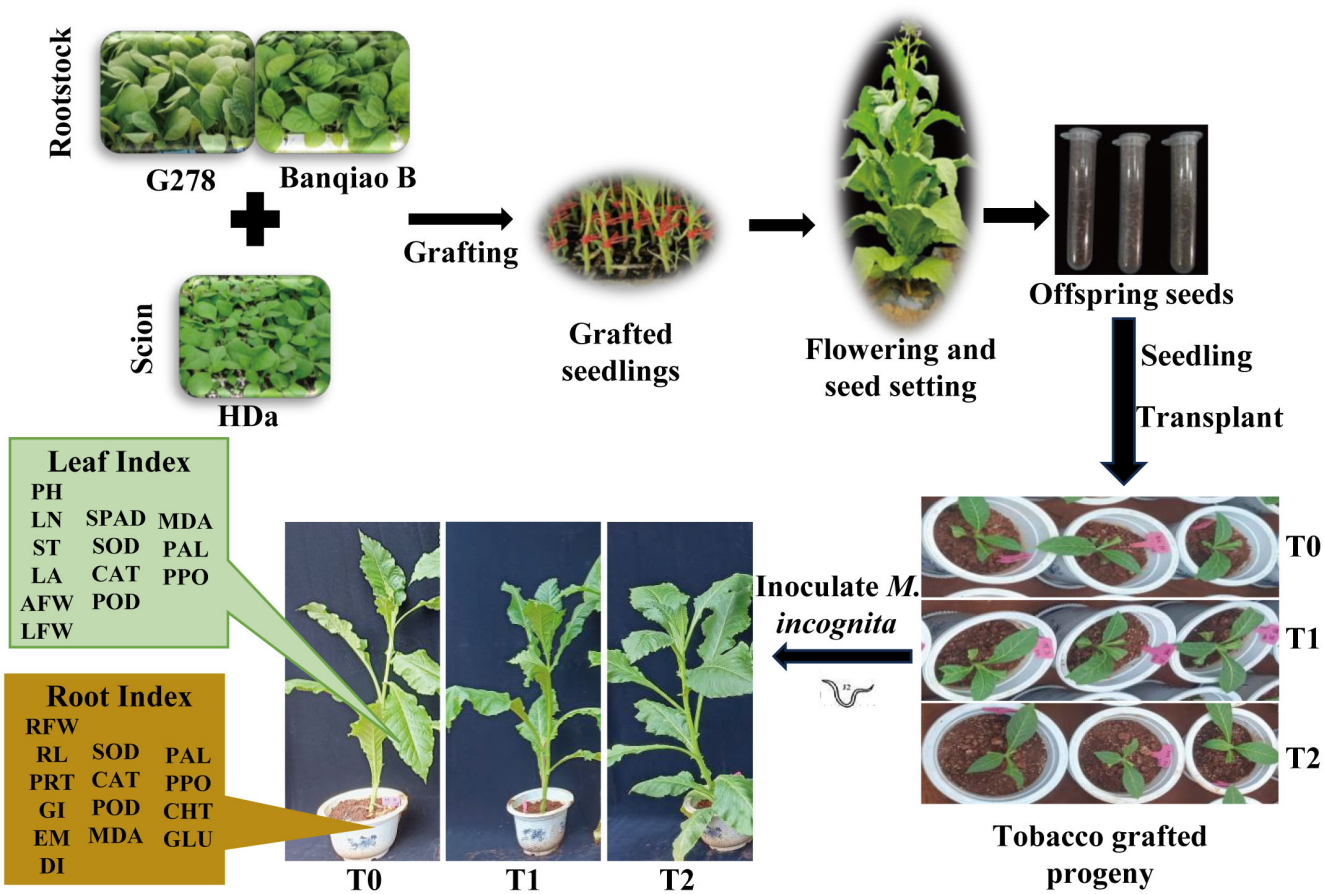
Experimental design of tobacco grafted progeny inoculated with root-knot nematodes. PH, Plant height; LN, Leaf number; ST, Stem diameter; LA, Leaf area; AFW, Aboveground fresh weight; LFW, Leaf fresh weight; RFW, Root fresh weight; RL, Root length; PRT, Percentage of root galls; GI, Gall index; EM, Egg masses; DI, Disease index. Same below.

From May to September 2023, a pot-experiment using tobacco grafted progeny was conducted in the greenhouse at Yunnan Agricultural University, Kunming, Yunnan Province, China (25°8′10.95″N, 102°45′14.59″E). The environmental conditions were maintained at a temperature range of approximately 17 - 32 °C and a relative humidity of around 67%. Planting pots measured 25 cm in diameter and 30 cm in height. The potting substrate consisted of red soil, humus, and perlite (4:4:1 ratio), sterilized at high temperature before use. When seedlings reached the 3–4 true-leaf stage, they were transplanted and inoculated. Each pot contained one seedling, with eight pots per variety constituting one replicate. Three independent replicates were maintained per treatment. All tobacco seedlings were inoculated with an RKN egg suspension. During inoculation, 50 mL of suspension (approximately 5,000 eggs; 10 mL egg concentrate + 40 mL distilled water) was applied evenly into a circular trench 1.0-1.5 cm from the stem base. The trench measured approximately 1.5 cm deep and wide. The suspension was uniformly applied along the outer edge of the tobacco roots, and then covered with soil. The pots were arranged randomly in the greenhouse, with uniform plant management to prevent pest interference or secondary infections. The experimental design is shown in **Figure 1**.

### 2.3 Determination items and methods

#### 2.3.1 Agronomic traits

At 30, 60, and 90 days post-inoculation, six tobacco plants per treatment were selected for measurement of plant height, stem diameter, number of effective leaves, and length and width of the largest middle leaf, with subsequent leaf area calculation (Equation 1). At 90 days, the fresh weights of the aerial parts, leaves, and roots, as well as root length, were measured for six tobacco plants.


(1) $$\text{Leaf area }\left({\text{cm}}^{2}\right)=\text{Leaf length}\times \text{Leaf width}\times 0.6345\;\left(\text{leaf area coefficient}\right)$$

#### 2.3.2 Disease resistance index

Referring to the method in the literature (Xu et al., 2024), at 90 days post-inoculation, root-knot percentage, number of root-knots, and number of egg masses were recorded for each treatment. The gall index (GI) (Equation 2) and Disease index (DI) (Equation 3) were calculated. DI quantifies plant health and disease severity (Wei et al., 2023) and was used to assess disease resistance (**Table 1**).

**Table 1:** Grading standards for tobacco root-knot nematode disease and classification criteria for resistance types.

Grade of disease	Disease severity	Disease index	Resistance type
0	No root-knots observed	0	Highly resistant or immune(I)
1	Root-knots occur on less than 1/4 of the roots	0.1-20	Resistant(R)
3	Root-knots occur on 1/4-1/3 of the roots	20.1-40	Moderately resistant(MR)
5	Root-knots occur on 1/3-1/2 of the roots	40.1-60.0	Moderately susceptible(MS)
7	More than half of the roots exhibited root-knots, with a few secondary roots also forming galls	60.1-80.0	Susceptible(S)
9	All roots, including secondary roots, exhibited root-knots	80.1-100	Highly susceptible(HS)

The assessment of disease severity, disease index, and resistance type primarily refers to two Chinese standards: “Grade and investigation method of tobacco diseases and insect pests” (GB/T 23222-2008) and “Identification of cultivar resistance to tobacco disease” (GB/T 23224-2008) (Website: https://std.samr.gov.cn/).


(2) GI=Total number of root knots per plant/Fresh root weight per plant

(3) DI=(∑(Mi×Si)/(N×9))×100%)

In the above equation, *Si* represents the severity value of disease level *i*, *Mi* represents the number of plants with disease level *i*, *i* represents the disease severity level, and *N* represents the total number of plants surveyed.

#### 2.3.3 Physiological and biochemical indicators

Soil and Plant Analyzer Development (SPAD) readings: At 90 days post-inoculation, six tobacco plants per treatment were selected between 8:00 and 10:00. SPAD-502 chlorophyll meter readings were taken at three positions on the main vein of the central leaf: base, middle, and tip (He et al., 2020).

Determination of pathogenesis related enzyme activities: Samples were collected at 90 days post-inoculation, with three pots per treatment. Roots and leaves were washed, dried with absorbent paper, and leaf veins were removed. The tissues were then cut into small pieces, mixed thoroughly, flash-frozen in liquid nitrogen, and stored in an ultralow-temperature freezer. Enzyme activities were measured using a multifunctional microplate reader (Varioskan LUX) and assay kits from Jiangsu Gresbio Biotechnology Co., Ltd. The following kits were employed: Superoxide Dismutase (SOD) Assay Kit (WST-8 method, units: U·g^-1^ FW), Catalase (CAT) Assay Kit (hydrogen peroxide method, units: umol·min^-1^·g^-1^ FW), Peroxidase (POD) Assay Kit (guaiacol method, units: △OD_470_·min^-1^·g^-1^ FW), Malondialdehyde (MDA) Content Assay Kit (Thiobarbituric acid colorimetric method, units: nmol·g^-1^ FW), Phenylalanine Ammonia-Lyase (PAL) Assay Kit (L-phenylalanine borate buffer method, units: △OD_290_·h^-1^·g^-1^ FW), Polyphenol Oxidase (PPO) Assay Kit (catechol method units: △OD_420_·min^-1^·g^-1^ FW), Chitinase (CHT) Assay Kit (units: ug·h^-1^·g^-1^ FW), and β-1,3-Glucanase (GLU) Assay Kit (units: ug·min^-1^·g^-1^ FW) (Ge et al., 2014; He et al., 2024). Procedures and calculations were performed following the manufacturer’s instructions.

### 2.4 Data analysis and statistics

Data analysis was performed using Microsoft Excel 2022 and DPS data analysis software. Duncan’s new multiple range test was employed for significance analysis, and graphs were generated using Origin 2017 software (Tong et al., 2024).

## 3 Results

### 3.1 Effects of RKN infection on agronomic traits

**Figure 2:**
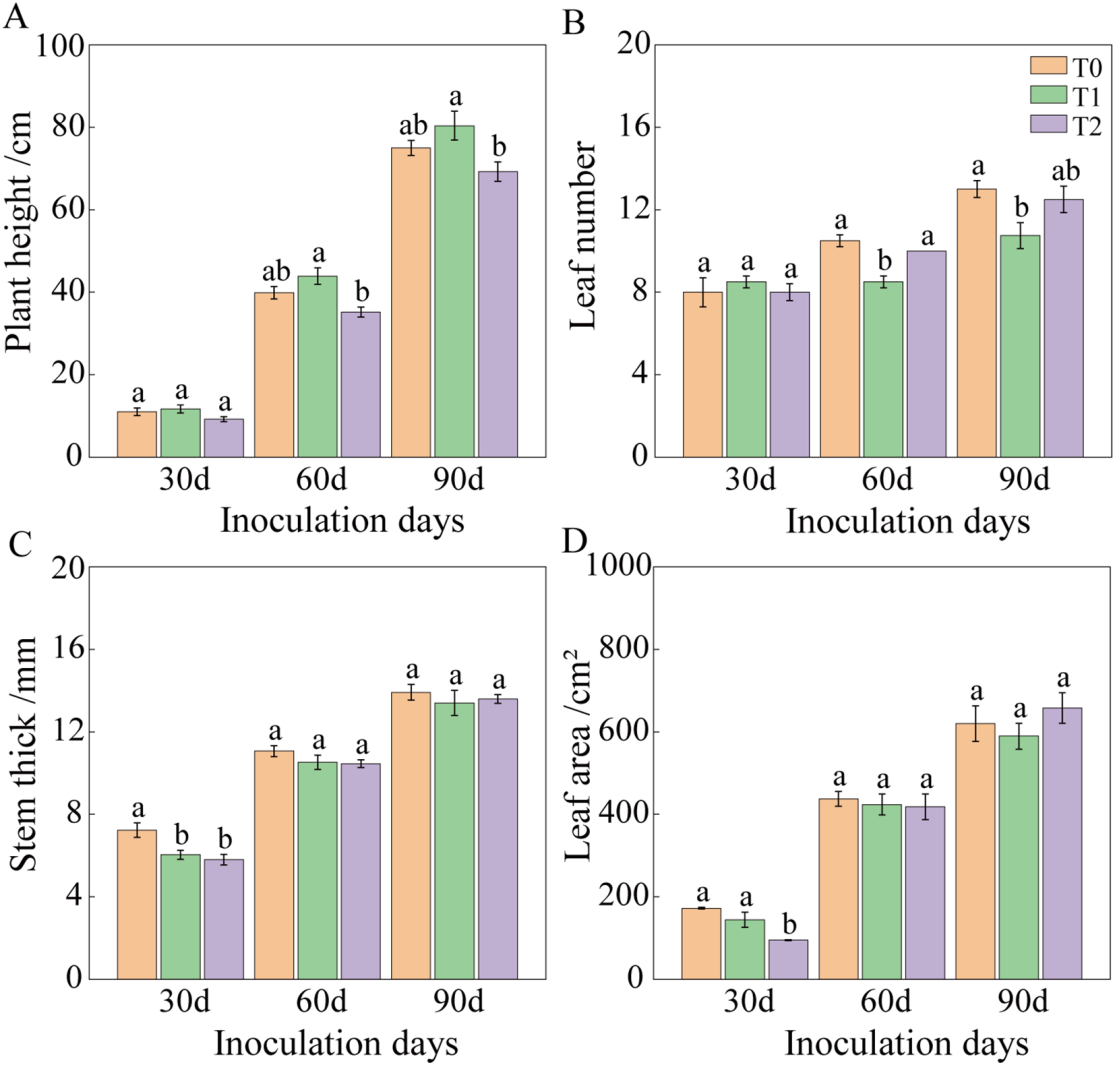
Agronomic traits of tobacco grafted progeny after root-knot nematode infection. **(A)** Plant height; **(B)** Leaf number; **(C)** Stem diameter; **(D)** Leaf area. Bars with different lowercase letters within the same group indicate significant differences between treatments (P< 0.05). Similar notation applies throughout. Lowercase letters “a” and “b” indicate significant differences between treatments. Identical letters indicate no significant difference between treatments, while different letters indicate a significant difference.

**Figure 3:**
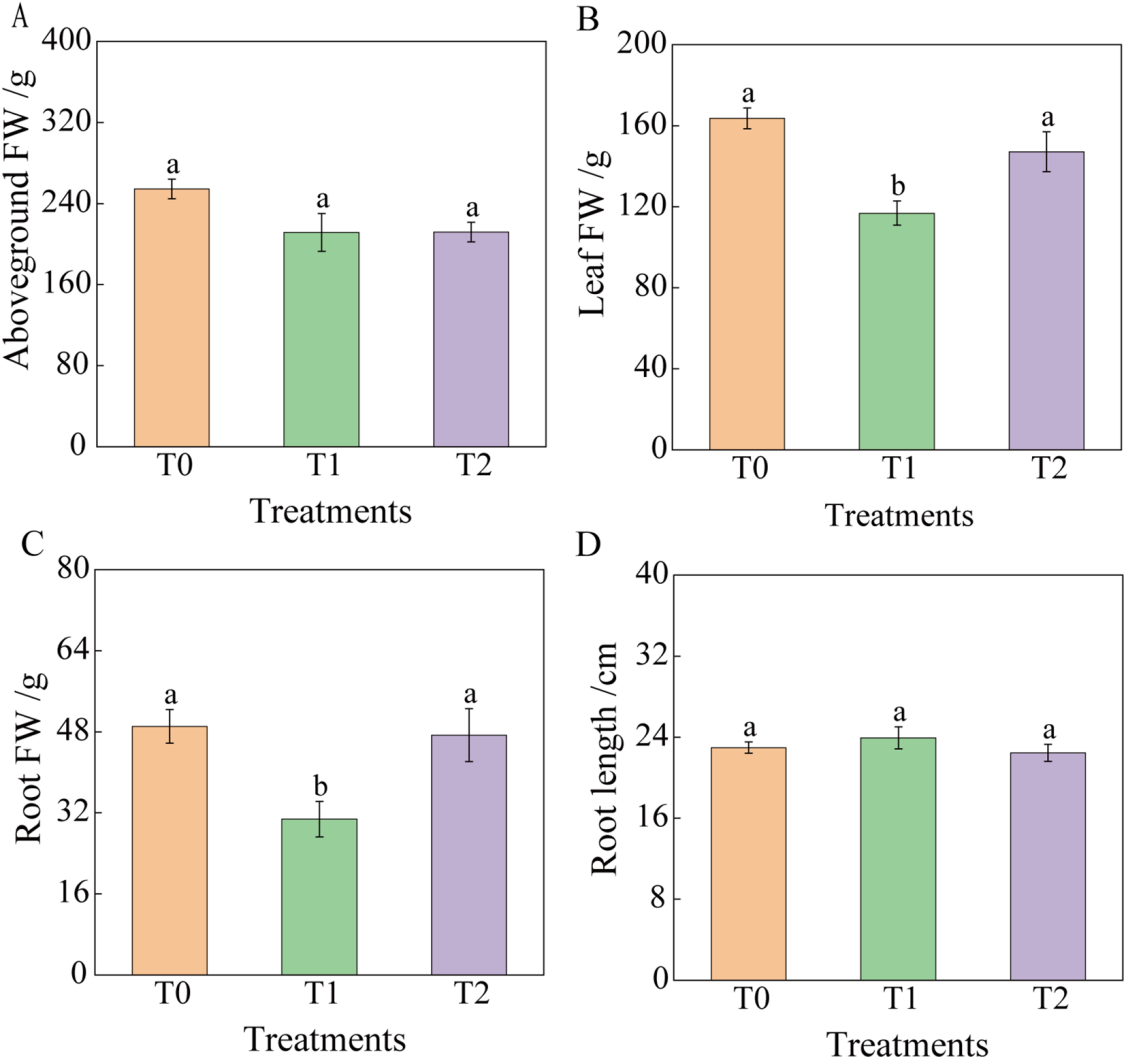
Effects of root-knot nematode infection on the fresh weight biomass of the aboveground part and roots of tobacco grafted progeny. **(A)** Aboveground fresh weight; **(B)** Leaf fresh weight; **(C)** Root fresh weight; **(D)** Root length, with significance set at p< 0.05. Lowercase letters “a” and “b” indicate significant differences between treatments. Identical letters indicate no significant difference between treatments, while different letters indicate a significant difference.

Following RKN inoculation, tobacco graft combination progeny exhibited altered agronomic traits (**Figures 2**, **3**). At 30 days post-inoculation, plant height and leaf number showed no significant differences among T0, T1, and T2. However, stem diameter in T1 and T2 decreased significantly by 16.61% and 19.86%, respectively, compared to T0. Leaf area of T2 was significantly reduced by 45.07% relative to T0, whereas T1 and T0 showed no significant difference. At 60 days post-inoculation, plant height of T2 decreased by 11.72% compared to T0, although not significantly. No significant difference occurred in plant height between T1 and T0, but leaf number of T1 was significantly lower than that of T2 and T0, whereas leaf number of T2 and T0 showed no significant difference. Stem diameter and leaf area exhibited no significant variations among treatments. By 90 days post-inoculation, no significant differences occurred among treatments in stem diameter, leaf area, aboveground fresh weight, or root length. Plant height differences between T0 and T1, as well as between T0 and T2, were not significant, but T1 and T2 differed significantly. Functional leaf number showed no significant difference between T0 and T2 or between T1 and T2, although T0 and T1 differed significantly. Fresh leaf and root weights showed no significant difference between T0 and T2, but T1 exhibited significantly lower values than both T0 and T2. At 90 days, overall growth appeared similar across tobacco graft progeny, with plant height ranking T2< T0< T1. Specifically, T2 exhibited a 7.60% and 13.88% reduction in plant height compared to T0 and T1, respectively. Leaf number of T1 was significantly lower than that of T0. Compared to T0 and T2, T1 showed significant reductions in fresh leaf weight (28.56% and 20.58%, respectively) and fresh root weight (37.29% and 34.99%, respectively).

In summary, agronomic traits of the tobacco graft combination progeny showed limited variation compared to T0. Specifically, T2 exhibited significantly reduced plant height, and T1 displayed significantly fewer functional leaves and lower biomass. No significant differences occurred in other measured traits.

### 3.2 Disease incidence

**Figure 4:**
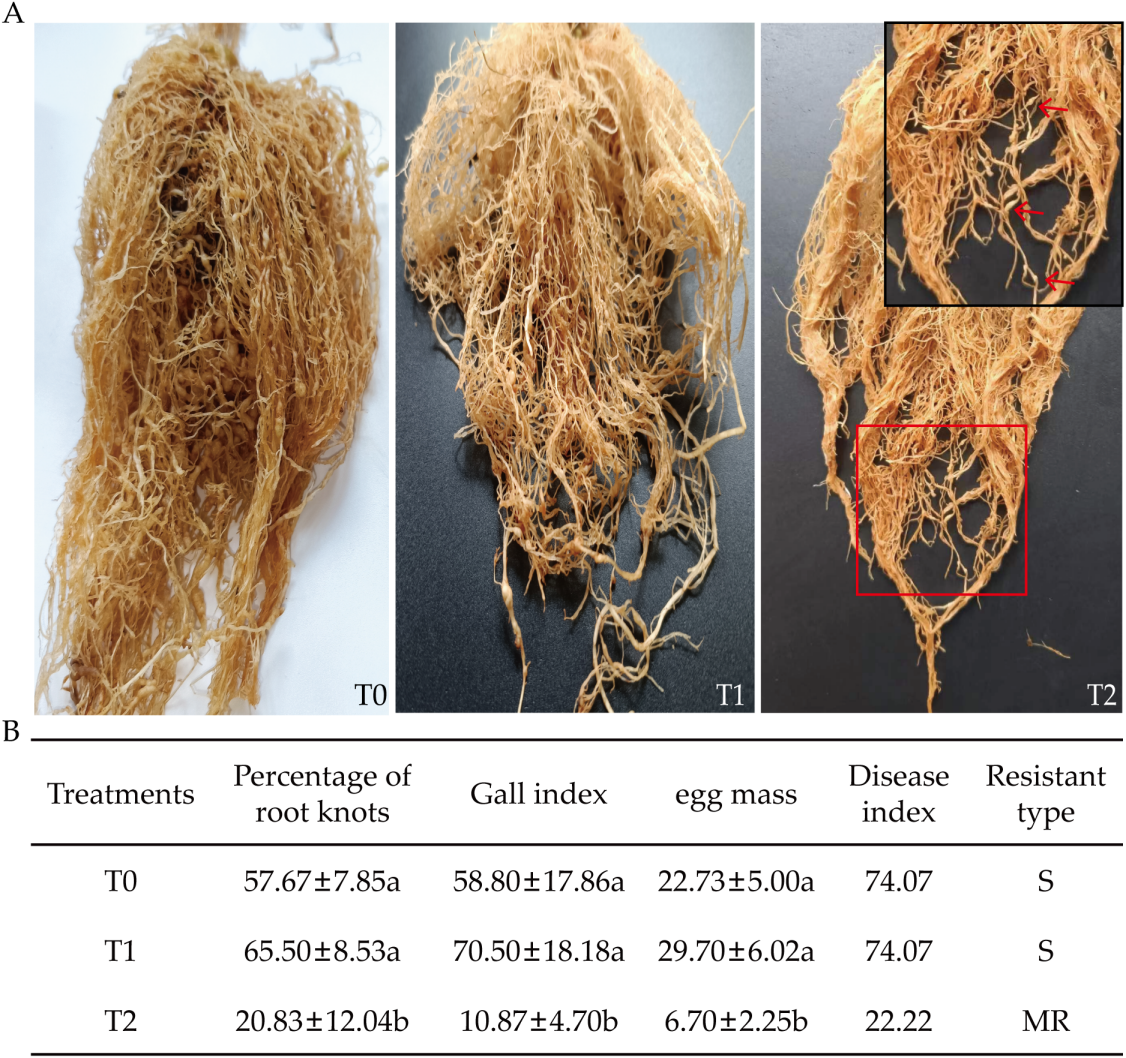
Effects of root-knot nematode infection on disease resistance-related indicators in tobacco grafted progeny. **(A)** Symptoms of disease in T0, T1, and T2 after RKN infection; **(B)** Disease resistance indicators in tobacco graft progeny after RKN infection, with significance set at p< 0.05. The red arrow indicates the root knot. Lowercase letters “a” and “b” indicate significant differences between treatments. Identical letters indicate no significant difference between treatments, while different letters indicate a significant difference.

After 90 days of RKN inoculation, grafted progeny and control exhibited differential RKN-induced damage (**Figure 4**). T0 and T1 showed extensive gall formation, while root growth was inhibited compared to uninfected plants. T2 displayed minimal damage with few visible galls (**Figure 4A**). At 90 days post-inoculation, significant differences occurred among T0, T1, and T2 in root-knot percentage, GI, egg mass count, DI, and incidence rate (**Figure 4B**). Compared to T0, T2 exhibited reductions of 63.88% in root-knot percentage, 81.51% in GI, and 71.82% in egg mass count. Relative to T1, T0 showed significant reductions of 68.19%, 84.58%, and 63.87% in these same parameters, respectively. DI was highest in T0 and T1 (both at 74.07), while T2 had the lowest value (22.22). Based on DI, T2 was classified as moderately resistant (MR), whereas T0 and T1 were susceptible (S). In summary, T2 exhibited the lowest values across all disease indicators, showing a high level of resistance to RKNs.

### 3.3 Effect of RKN infestation on the SPAD values of tobacco leaves

**Figure 5:**
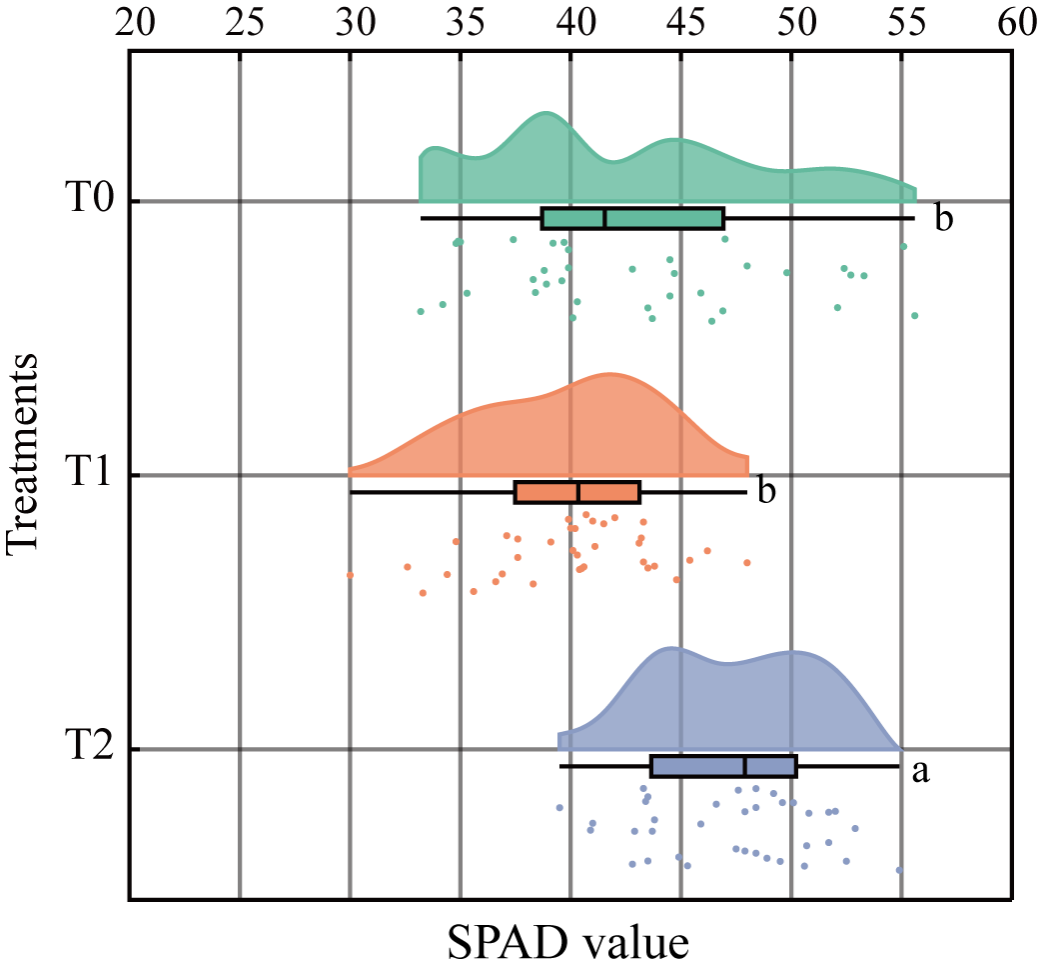
Effects of root-knot nematode infection on the tobacco grafting combinations on the SPAD value of leaves (P< 0.05). Lowercase letters “a” and “b” indicate significant differences between treatments. Identical letters indicate no significant difference between treatments, while different letters indicate a significant difference.

At 90 days post-inoculation with RKN, SPAD values in T1 and T0 showed no significant difference, whereas T2 exhibited significantly higher values than T0 (**Figure 5**). This suggests that SPAD values in tobacco graft progeny responded differently following nematode infection. Overall SPAD ranking was T2 > T0 > T1. SPAD value decreased by 7.12% in T1 relative to T0, but increased by 10.03% in T2 relative to T0 and 18.47% relative to T1. These results demonstrate that nematode-resistant T2 progeny showed the greatest SPAD elevation, reflecting increased chlorophyll content. Resistant progeny (T2) also displayed higher SPAD values than the susceptible scion (HD).

### 3.4 Effects of RKN infection on antioxidant enzyme activities and MDA content

**Figure 6:**
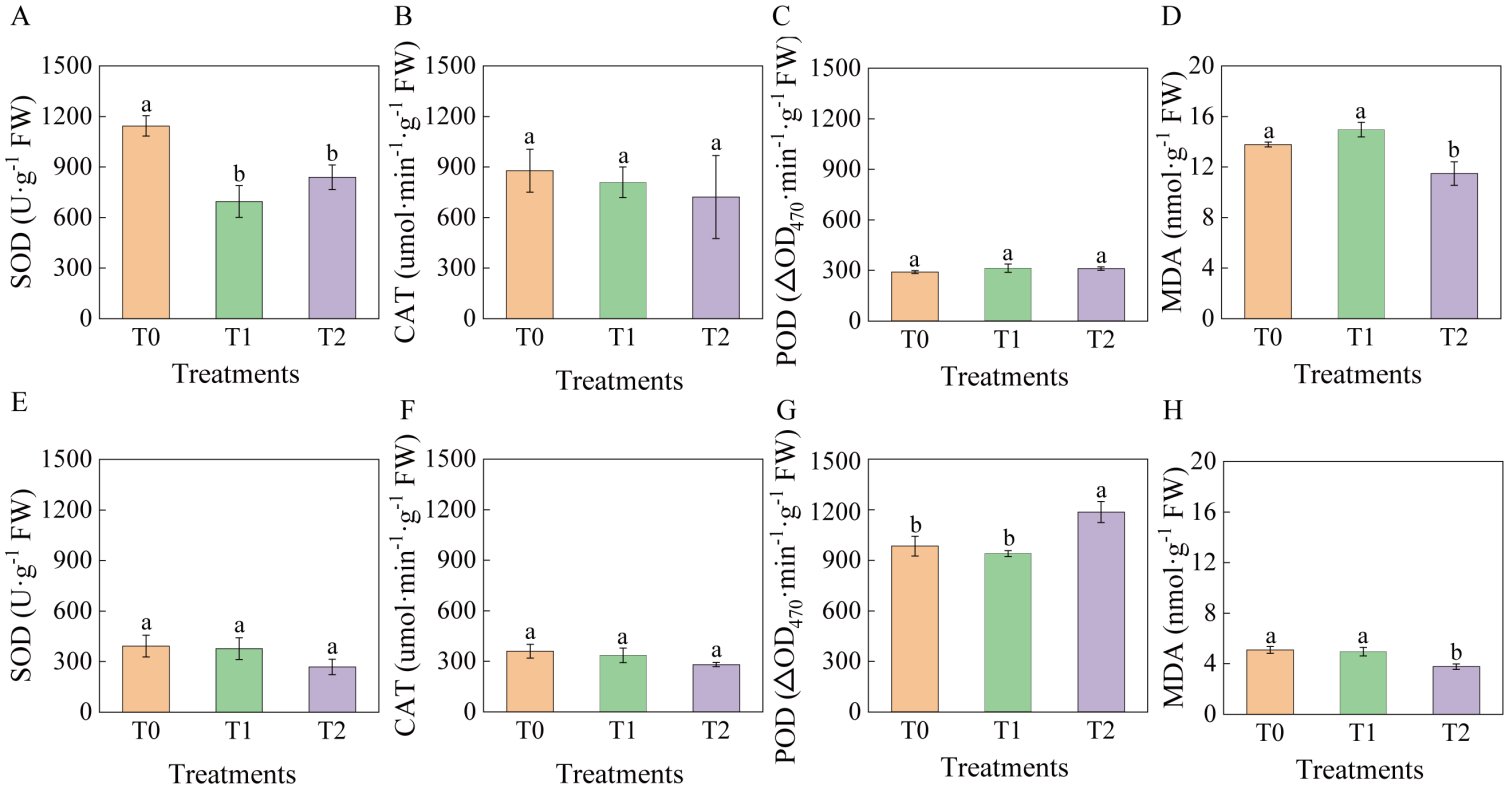
Effects of root-knot nematode infection on antioxidant enzyme activities and MDA content in grafted tobacco progeny. **(A)** Leaf SOD activity; **(B)** Leaf CAT activity; **(C)** Leaf POD activity; **(D)** MDA content in leaves; **(E)** Root SOD activity; **(F)** Root CAT activity; **(G)** Root POD activity; **(H)** MDA content in roots, with significance set at p< 0.05. Lowercase letters “a” and “b” indicate significant differences between treatments. Identical letters indicate no significant difference between treatments, while different letters indicate a significant difference.

At 90 days post-infection with RKN, activities of SOD, CAT, and POD, as well as MDA content in leaves and roots of tobacco graft progeny, varied relative to T0 (**Figure 6**). Leaf SOD activity in T1 and T2 decreased significantly by 39.21% and 26.65%, respectively, compared to T0 (**Figure 6A**), while root SOD showed no significant change (**Figure 6E**). CAT activity in both leaves and roots exhibited no significant differences compared to T0 (**Figures 6B, F**). Leaf POD activity showed no significant differences among treatments, whereas root POD activity in T2 increased significantly by 20.40% relative to T0 and 26.15% relative to T1 (**Figures 6C, G**). MDA content in T2 leaves and roots was significantly lower than T0 (16.58% and 25.92%, respectively) and T1 (23.13% and 23.88%, respectively); no significant difference occurred between T1 and T0 (**Figure 6D, H**).

Overall, graft progeny exhibited lower root SOD/CAT activities and MDA content but higher root POD activity than corresponding leaves. T2 exhibited enhanced resistance, elevated antioxidant responses, and reduced lipid peroxidation, indicating diminished membrane damage under nematode stress.

### 3.5 Effects of RKN infection on phenylpropanoid metabolic enzymes and pathogenesis related proteases in tobacco

**Figure 7:**
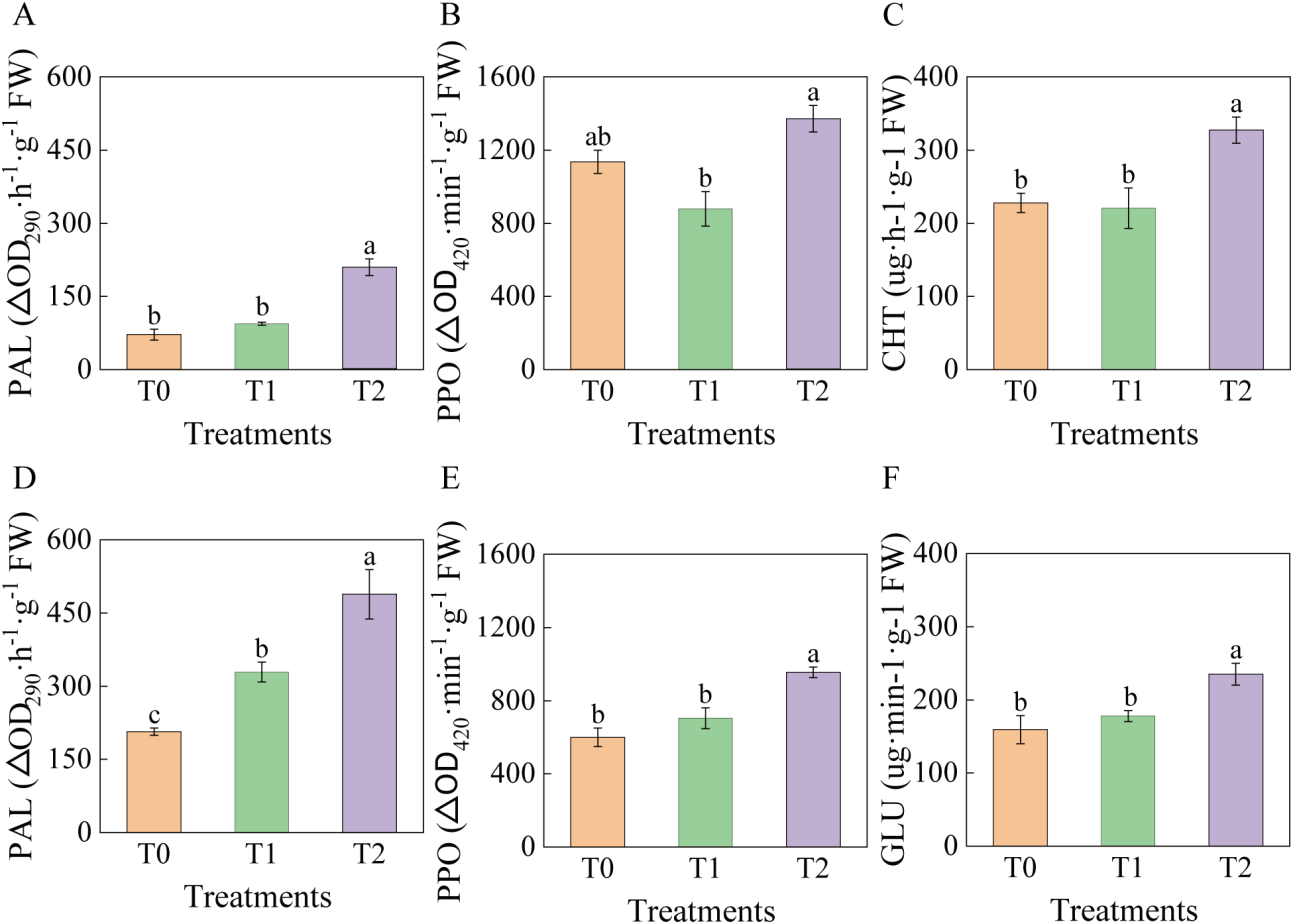
Effects of root-knot nematode infection on phenylpropanoid metabolic enzymes and pathogenesis-related protease activities in tobacco graft progeny. **(A)** Represents the PAL activity of the leaves; **(B)** Represents the PPO activity; **(C)** CHT activity in roots; **(D)** Represents the PAL activity of the roots; of the leaves; **(E)** Represents the PPO activity of the roots; **(F)** GLU activity in roots, with significance set at p< 0.05. Lowercase letters “a” and “b” indicate significant differences between treatments. Identical letters indicate no significant difference between treatments, while different letters indicate a significant difference.

At 90 days post-inoculation, significant changes occurred in PAL and PPO activities in leaves and roots of tobacco grafted progeny (**Figure 7**). The highest PAL activity was recorded in T2 leaves and roots, increasing significantly by 192.12% and 136.31% compared to T0, and by 123.64% and 48.46% relative to T1 (**Figure 7A, D**). Among grafted progeny, leaf PPO activity in T2 was significantly higher than in T1 (56.14% increase) but not significantly different from T0. No significant differences were observed between T1 and T0 (**Figure 7B**). Root PPO activity in T2 increased significantly by 59.29% relative to T0 and 35.69% relative to T1, with T1 also showing significant increase relative to T0 (**Figure 7E**). CHT and GLU activities in T2 roots were significantly higher than both T0 and T1, whereas T1 and T0 showed no significant differences (**Figure 7C, F**).

In summary, phenylpropanoid enzymes and pathogenesis-related proteins showed significantly elevated activities in T2, reflecting enhanced defense responses against RKN infection.

### 3.6 Correlation analysis

**Figure 8:**
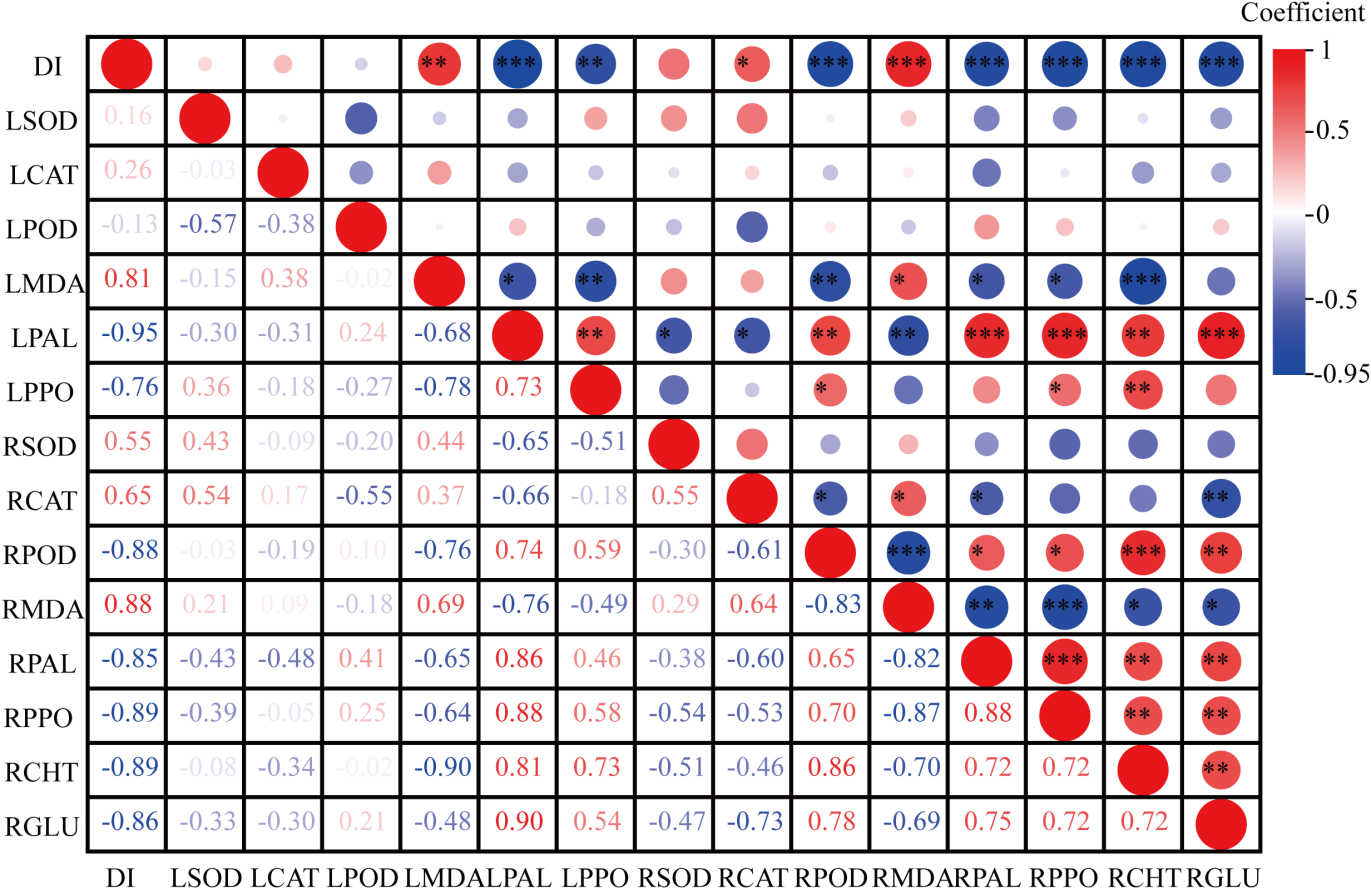
Correlation between biochemical indicators and disease index in the tobacco grafted progeny. “L” represents the biochemical indicators of leaves, and “R” represents the biochemical indicators of roots. The same applies below. *, **, and *** indicate statistically significant correlations at the levels of p< 0.05, p< 0.01, and p< 0.001, respectively.

The disease resistance of tobacco grafted progeny is a complex trait resulting from the coordinated response of multiple tissues and physiological pathways, which cannot be reliably assessed through single indicators. Consequently, correlation and principal component analyses (PCA) were performed to evaluate associations among physiological and resistance-related traits. Results (**Figure 8**) revealed that DI exhibited strong negative correlations with LPAL (r=-0.95), LPPO (r=-0.76), RPOD (r=-0.88), RPAL (r=-0.85), RPPO (r=-0.89), RCHT (r=-0.89), and RGLU (r=-0.86), while showing positive correlations with LMDA (r = 0.81), RCAT (r = –0.65), and RMDA (r = –0.88).

**Figure 9:**
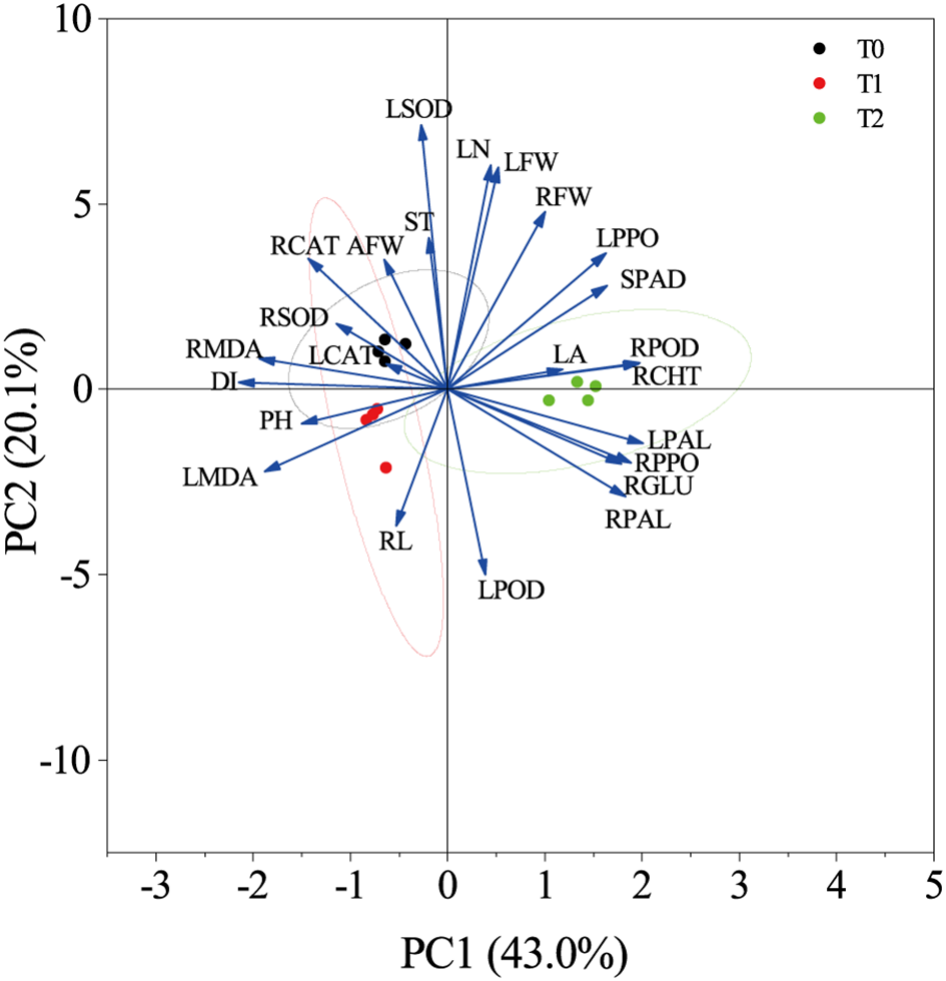
Principal component analysis of various indicators under root-knot nematode stress.

PCA (**Figure 9**) revealed that the first two principal components together accounted for 63.1% of the total variance. DI was located in the second quadrant and showed negative correlations with SPAD, LPOD, LPPO, LPAL, RCHT, RGLU, RPOD, RPAL, and RPPO in quadrants one and four. T1 and T2 were distributed in distinct PCA regions from the control T0, with tighter clustering of biological replicates. T0 localized to quadrant two, T1 to quadrant three, and T2 to quadrants one and four, further demonstrating clear phenotypic divergence between T2 and T0. These findings indicate that T2 exhibits increased SPAD values and enhanced activity of key defense enzymes under RKN stress, thereby improving resistance to nematode invasion—confirming its higher resistance phenotype relative to susceptible control T0.

## 4 Discussion

Grafting techniques are widely applied to vegetable crops such as eggplants, tomatoes, cucumbers, and melons to improve propagation efficiency, varietal preservation, yield, stress tolerance, and fruit quality (Sigüenza et al., 2005; Kokalis-Burelle and Rosskopf, 2011; Judy et al., 2013). Research has primarily focused on the effects of rootstocks and scions in grafted plants, with limited attention to heritable genetic effects in grafted progeny (Mudge et al., 2009). Here, we demonstrate that RKN-resistant rootstocks significantly influence progeny phenotypes, suggesting stable inheritance through sexual reproduction. Progeny from tobacco graft combinations with resistant rootstocks retained rootstock disease-resistant traits. Compared to susceptible controls, the progeny exhibited enhanced antioxidant activities, elevated phenylpropanoid pathway enzymes, and increased defense-related proteins, improving resistance against RKNs.

Grafting can induce heritable genetic variation and enhance growth performance in plant progeny (Hardik et al., 2020). Studies have shown that progeny exhibit significantly enhanced vigor and seed yield when using Arabidopsis MSH1 mutants as rootstocks for tomato grafts (Hardik et al., 2020). Similarly, when tomato was used as rootstocks for *Solanum americanum*, progeny showed markedly increased aboveground biomass (Wang et al., 2021b). When tobacco roots are infected by RKNs, root galls form, cells are damaged, and giant cells develop, ultimately impairing normal tobacco growth (Zhang et al., 2023). Our results show that GHF1 growth traits showed no significant difference from susceptible scion HD, suggesting mitigation of nematode-induced growth suppression. This finding diverges from previous reports, which may reflect limited transgenerational phenotypic variation in GHF1. Notably, GHF1 exhibited a moderately resistant phenotype significantly superior to susceptible HD. This suggests that progeny derived from crosses between resistant rootstock G278 and susceptible scion HD can inherit partial nematode resistance, representing the first documented case of nematode resistance transmission through tobacco grafting progeny.

Under RKN stress, plants exhibit reduced photosynthetic efficiency and stomatal conductance, disrupting nutrient uptake, photosynthate synthesis, and transport regulation in roots (Mujeebur et al., 2022). These physiological disruptions reduce chlorophyll accumulation. Chlorophyll reduction serves as a key physiological stress indicator (Zhu et al., 2023). Given the strong correlation between chlorophyll content and SPAD values, SPAD readings provide a reliable chlorophyll proxy (Ling et al., 2011). Studies have shown that resistant soybean cultivars maintain higher chlorophyll levels than susceptible cultivars under RKN stress (Gontijo et al., 2024). Previous studies have also indicated that grafting can enhance photosynthetic pigments in plants under stress (Du et al., 2024). Consistent with these reports, PCA revealed a negative correlation between SPAD values and DI, and disease-resistant GHF1 showed significantly higher chlorophyll content than susceptible BHF1 and scion HD. This suggests that chlorophyll accumulation is enhanced in resistant progeny compared to susceptible controls, supporting improved photosynthetic performance. Therefore, grafting may promote chlorophyll biosynthesis in graft-derived progeny under nematode stress.

Numerous studies demonstrate that antioxidant enzyme systems crucially maintain ROS homeostasis in plants. Both grafted and self-rooted tomato seedlings exhibited induced antioxidant enzyme activities (Bali et al., 2020). Scion leaves of grafted plants showed significantly higher SOD, POD, and CAT activities than self-rooted plants, with enhanced ROS-scavenging capacity (Liang et al., 2012). Increased POD activity is typically an indicator of enhanced disease resistance in plants (Gontijo et al., 2024). In resistant tomato plants, root and leaf SOD activity decreases post-inoculation, while POD and CAT exhibit minimal initial changes followed by rapid POD increase (Jia and Xu, 2016). Consistent with these reports, GHF1 exhibited significantly elevated root POD activity but SOD and CAT levels comparable to HD in this study. In leaves, SOD activity decreased in GHF1, while CAT and POD remain stable. This pattern may be associated with lower SOD activity during later infection stages, which helps sustain localized ROS accumulation, potentially enhancing the hypersensitive response to RKN infection. Increased POD activity may also promote cell wall lignification, improving resistance to secondary infections (Wang et al., 2009). Under stress, plants often undergo membrane lipid peroxidation, with malondialdehyde (MDA) content reflecting the extent of lipid peroxidation and cellular damage (Guan et al., 2016). Studies have shown that MDA levels in grafted cucumber seedlings are consistently reduced relative to self-rooted controls (Tian et al., 2012). In this study, MDA content in both leaves and roots of GHF1 was significantly reduced compared to HD. The altered antioxidant enzyme profiles in graft-derived progeny suggest a potential heritable shift in antioxidant capacity, although underlying mechanisms require further elucidation (Wang et al., 2021a).

Phenylalanine ammonia-lyase exhibits significantly increased activity in response to pathogen infection and contributes to plant defense by regulating the biosynthesis of lignin and phenolic compounds (Ganapathy et al., 2016). Polyphenol oxidase and peroxidase act synergistically to catalyze the formation of toxic quinones, which in turn inhibit the activity of pathogen-associated enzymes in phytopathogenic microorganisms (Jung et al., 2004). In the grafting system using *Solanum torvum* as rootstock and *Solanum melongena* as scion, PAL and PPO activities in scion leaves were significantly higher post-RKN inoculation compared to self-rooted plants (Wang et al., 2008). Enhanced PAL and PPO activities in grafted pepper plants showed a significant correlation with improved cellular-level disease resistance (Naik et al., 2024). Consistent with these reports, PAL and PPO activities showed significant negative correlations with the DI in this study. GHF1 grafted progeny exhibited significantly higher PAL and PPO activities in both leaves and roots than scion HD, aligning with its enhanced RKN resistance observed here and previously reported. Chitinase degrades chitin in nematode eggshells and body walls, while GLU exerts nematocidal effects by inducing phytoalexin synthesis (Ahuja et al., 2012; Li et al., 2020). Resistant eggplant rootstocks showed significantly higher CHT and GLU activities than susceptible varieties post-RKN inoculation (Xu et al., 2008). Here, CHT and GLU activities in GHF1 roots were significantly elevated relative to HD following RKN infection. Principal component analysis further revealed significant positive correlations between these enzyme activities and RKN resistance.

## 5 Conclusion

Following RKN infection, the grafted progeny GHF1 exhibited markedly enhanced resistance compared to its scion HD, characterized by elevated chlorophyll levels, increased activity of phenylpropanoid metabolic enzymes and disease-related proteins, reduced membrane lipid peroxidation, and stable antioxidant enzyme levels. Among all combinations tested, GHF1 displayed the most robust resistance phenotype, underscoring its potential as a superior germplasm resource. By integrating phenotypic, physiological, and biochemical analyses, this study provides a framework for evaluating the agronomic performance and stress responses of graft-derived tobacco progeny under nematode pressure. These findings not only highlight the promise of grafting in enhancing disease resistance but also raise important questions about the stability and heritability of such traits across generations. Future investigations should aim to unravel the genetic architecture underlying transgenerational resistance conferred by grafting, thereby advancing both fundamental understanding and applied tobacco breeding strategies.

## Data Availability

The raw data supporting the conclusions of this article will be made available by the authors, without undue reservation.
